# Random Mutagenesis Reveals Residues of JAK2 Critical in Evading Inhibition by a Tyrosine Kinase Inhibitor

**DOI:** 10.1371/journal.pone.0043437

**Published:** 2012-08-16

**Authors:** Michael R. Marit, Manprit Chohan, Natasha Matthew, Kai Huang, Douglas A. Kuntz, David R. Rose, Dwayne L. Barber

**Affiliations:** 1 Department of Medical Biophysics, Faculty of Medicine, University of Toronto, Toronto, Ontario, Canada; 2 Campbell Family Cancer Research Institute, Ontario Cancer Institute, Toronto, Ontario, Canada; 3 Department of Biology, University of Waterloo, Waterloo, Ontario, Canada; Institut National de la Santé et de la Recherche Médicale, France

## Abstract

**Background:**

The non-receptor tyrosine kinase JAK2 is implicated in a group of myeloproliferative neoplasms including polycythemia vera, essential thrombocythemia, and primary myelofibrosis. JAK2-selective inhibitors are currently being evaluated in clinical trials. Data from drug-resistant chronic myeloid leukemia patients demonstrate that treatment with a small-molecule inhibitor generates resistance via mutation or amplification of *BCR-ABL*. We hypothesize that treatment with small molecule inhibitors of JAK2 will similarly generate inhibitor-resistant mutants in JAK2.

**Methodology:**

In order to identify inhibitor-resistant JAK2 mutations *a priori*, we utilized TEL-JAK2 to conduct an *in vitro* random mutagenesis screen for JAK2 alleles resistant to JAK Inhibitor-I. Isolated mutations were evaluated for their ability to sustain cellular growth, stimulate downstream signaling pathways, and phosphorylate a novel JAK2 substrate in the presence of inhibitor.

**Conclusions:**

Mutations were found exclusively in the kinase domain of JAK2. The panel of mutations conferred resistance to high concentrations of inhibitor accompanied by sustained activation of the Stat5, Erk1/2, and Akt pathways. Using a JAK2 substrate, enhanced catalytic activity of the mutant JAK2 kinase was observed in inhibitor concentrations 200-fold higher than is inhibitory to the wild-type protein. When testing the panel of mutations in the context of the *Jak2* V617F allele, we observed that a subset of mutations conferred resistance to inhibitor, validating the use of TEL-JAK2 in the initial screen. These results demonstrate that small-molecule inhibitors select for *JAK2* inhibitor-resistant alleles, and the design of next-generation JAK2 inhibitors should consider the location of mutations arising in inhibitor-resistant screens.

## Introduction

Myeloproliferative neoplasms (MPNs) are diseases characterized by an excess production of one or more fully differentiated blood cell types, and can be precursors to more severe disorders including myelodysplastic syndrome and acute leukemia [1], [2], [3]. Philadelphia chromosome-negative MPNs include polycythemia vera (PV), essential thrombocythemia (ET), and primary myelofibrosis (PMF).

The identification of a somatic valine to phenylalanine mutation at residue 617 of JAK2 was made in 90% of PV, 50% of ET, and 50% of PMF patients [4], [5], [6], [7]. JAK2 is a cytoplasmic tyrosine kinase that is constitutively associated with members of the cytokine receptor superfamily. Ligation of the receptor results in JAK2 cross-phosphorylation and activation of downstream pathways including the STAT family of transcription factors, the PI3-kinase/Akt survival pathway, and the ERK kinase pathway. Induction of these pathways results in transcription of genes required for survival and differentiation. The JAK2 V617F mutation lies in a domain previously thought to be a non-functional kinase domain. Recent work has demonstrated this ‘pseudo-kinase’ domain to be a functional dual-specificity kinase important in the negative regulation of cytokine signaling through phosphorylation of JAK2 Y570 and S523 [8]. Presence of the V617F mutation was demonstrated to reduce phosphorylation on Y570 and S523, residues important in maintaining a low level of activity in the JAK2 kinase domain. The JAK2 V617F mutation is thought to relieve the negative regulatory role of the dual-specificity kinase domain and is thus is weakly oncogenic, able to transform specific cell lines to cytokine independence [9].

Chronic myeloid leukemia (CML) is a Philadelphia chromosome-positive MPN characterized by the presence of the t(9;22)(q34;q11) chromosomal translocation [10] and the consequent expression of the BCR-ABL fusion protein [11]. Treatment of CML was revolutionized in 2001 with the development of the small-molecule inhibitor imatinib mesylate (IM) [12], [13], [14], which binds to the BCR-ABL kinase domain and that prevents its ability to phosphorylate target substrates [12], [15]. Patients generally respond very well to IM, demonstrating results ranging from a partial hematologic response to complete cytogenetic remission [13], [16]. However, inhibitor resistance-based patient relapse occurs due to amplification of the *BCR-ABL* fusion gene or a mutation in the kinase domain that prevent small-molecule inhibitor binding [17], [18], [19], [20]. In order to model BCR-ABL mutant generation, a BCR-ABL/IM *in vitro* system was developed to identify IM-resistant mutations [21], [22]. The resulting mutation spectrum bears a striking overlap with clinical results [22]. As such, the isolated mutations can be used to design next-generation inhibitors. Patients expressing small-molecule inhibitor-resistant mutations progress to next-generation inhibitors with variable results, largely depending on the specific mutation present [23], [24]. Notably, the BCR-ABL T315I mutation is highly resistant to most ATP-competitive inhibitors against which it was tested [20], [25], while many other IM-resistant mutations are susceptible to inhibition by second-generation inhibitors such as dasatinib [26]. These data suggest that both inhibitor-specific and ATP competitor-specific mutations can arise in response to drug treatment. Promising new inhibitors targeting different aspects of the BCR-ABL protein function are currently under development [27], [28], [29].

Discovery of JAK2 V617F and its role in PV, ET, and PMF started the search for a small-molecule inhibitor for JAK2. More than a dozen inhibitors have since been identified to reduce JAK2 V617F kinase activity *in vitro* [30], some of which are being tested in clinical trials [31], [32], [33]. To date, no inhibitor-resistant JAK2 mutations have been identified in patients. However, as JAK2 inhibitors become more widely used, we anticipate a relapse rate that approximates the results observed with IM. We hypothesize that this relapse may be due mutations in the JAK2 kinase domain that prevent inhibitor binding, as is the case with IM-treated BCR-ABL. Using a random mutagenesis approach, we have identified JAK2 kinase domain residues critical in evading small-molecule inhibition. Here we describe the identification and characterization of mutations in the JAK2 kinase domain that confer resistance to the presence of small-molecule inhibitors *in vitro*.

## Materials and Methods

### Antibodies

The anti-phosphotyrosine antibody 4G10, anti-ERK1/2, and anti-STAT5a/b antibodies were purchased from Upstate Biotechnology (Lake Placid, NY). The anti-phospho-ERK1/2 (pY204) and anti-GST antibodies were purchased from Santa Cruz Biotechnology (Santa Cruz, CA). The anti-phospho-STAT5 (pY694) antibody was purchased from Zymed (Carlsbad, CA). The anti-JAK2, anti-phospho-S6 (pS235/236), anti-S6, anti-phospho-Akt (pS473), anti-Akt antibodies were purchased from Cell Signaling (Beverly, MA). The horseradish peroxidase (HRP)-conjugated protein A, and donkey anti-rabbit-HRP IgG, sheep anti-mouse-HRP IgG antibodies were purchased from GE Healthcare UK (Little Chalfont, Buckinghamshire, UK).

### Plasmids

Human TEL-JAK2(5-12) and full-length murine Jak2 were cloned into the retroviral expression vector pMPG2. TEL-JAK2(5-12) contains the PNT dimerization domain of TEL fused to the kinase and pseudokinase domains of JAK2. The indicated point mutations in TEL-JAK2 and Jak2 were induced using the QuikChange site-directed mutagenesis kit (Stratagene; Santa Clara, CA). The JAK2 substrate was modeled after the activation loop of JAK2 (PQDKEYYKVKE; referred to as KEYY) and cloned into pEBG-GST in order to express a GST fusion protein. KEYY, KEYF, KEFY and KEFF were used in substrate optimization experiments with TEL-JAK2. KEYF was used to test the phosphorylation ability of TEL-JAK2 and its associated mutations, whereas KEYY was utilized to test the kinase activity of Jak2 V617F and its associated mutations. KEFF was used as a negative control.

### Inhibitors

JAK Inhibitor-I was purchased from EMD Chemicals (Gibbstown, NJ). CEP-701 (Lestaurtinib) was purchased from LC Laboratories (Woburn, MA). TG101348 was kindly donated by Ross Levine, Memorial Sloan-Kettering Cancer Center, New York, NY.

### Cell Lines and Cell Culture

BaF3 cells were cultured in RPMI 1640 medium supplemented with 10% heat-inactivated fetal calf serum, 50 nM β-mercaptoethanol (Thermo Fisher Scientific; Waltham, MA), and 10% WEHI-conditioned medium. BaF3-EPO-R cells were cultured in RPMI 1640 medium supplemented with 10% heat-inactivated fetal calf serum, 50 nM β-mercaptoethanol, and 0.5 units/mL of human recombinant erythropoietin (EPO). HEK-293T and the HEK-293T-based Phoenix cells were cultured in Dulbecco’s Modified Eagle’s Medium H21 supplemented with 10% heat-inactivated fetal calf serum. All cells were incubated at 37°C with 5% CO_2_.

### HEK-293T and Phoenix Cell Transfection

Cells were transfected with Lipofectamine 2000 (Invitrogen; Carlsbad, CA), according to the manufacturer’s instructions, and 0.1 µg of pEBG and/or 1.0 µg of pMPG2, unless otherwise indicated.

### BaF3 Cell Transduction

BaF3 cells were transduced using the Phoenix cell system as previously described [34].

### Random Mutagenesis and JAK2 Mutant Screen

pMPG2-TEL-JAK2(5-12) was used to transform XL1-Red Competent *E. coli* (Stratagene). A large volume of mutagenized plasmid was isolated from the XL1-Blue strain using a Maxiprep kit (Qiagen; Hilden, Germany). BaF3 cells were cultured and transduced with the mutagenized pMPG2-TEL-JAK2 library (as above). Transduced BaF3 cells were selected in cytokine-free RPMI medium for three days. Cells were then plated at a low concentration in soft agar containing cytokine-free medium plus 1.93 µM JAK Inhibitor-I. Colonies were then isolated and grown in cytokine-free RPMI containing 2.5 µM JAK Inhibitor-I. DNA was isolated using a mammalian genomic DNA extraction protocol. The TEL-JAK2 kinase and pseudokinase domains were sequenced to identify mutations.

### Cell Lysis

HEK-293T cells were gently washed with magnesium and calcium-free phosphate-buffered saline (-MgCl_2_ -CaCl_2_ PBS). Cells were washed and resuspended in 200 µL lysis buffer (1 M Tris-HCl pH 8.0; 4 M NaCl; 4% Triton X-100; 0.5 M EDTA; 0.5 M Na_4_P_2_O_7_; 0.5 M NaF; 0.08 M Na_3_VO_4_; 0.5 M PMSF; one complete protease inhibitor cocktail tablet (Roche; Mannheim, Germany)). BaF3 cells were washed once with Hank’s balanced salt solution buffered with 10 mM HEPES and resuspended in 200 µL lysis buffer (as above). Cell lysates were incubated on ice and cell debris was pelleted.

### GST In Vitro Mixing

HEK-293T cells expressing both pMPG2 and pEBG (Addgene plasmid 2227) were lysed (as above). Glutathione Sepharose 4B beads (GE Healthcare) were added to the cell lysis solution and incubated overnight with agitation at 4°C. The beads were then washed three times with PBS, and 50 µL of 1×sample buffer (as above, diluted 1∶1) was added prior to SDS-PAGE.

### SDS-PAGE and Immunoblot

Cell lysis and GST pull-down samples were resolved by sodium dodecyl sulfate–polyacrylamide gel electrophoresis (SDS-PAGE) and transferred to a polyvinylidene difluoride (PVDF) membrane. Western blotting was performed as previously described [35].

### XTT Assay

In order to quantify resistance conferred by specific TEL-JAK2 and Jak2 V617F mutations, an XTT assay was performed. All XTT experiments were performed in 96-well plates (Nunc) at an initial concentration of 2×10^3^ cells/well. BaF3 and BaF3-EPO-R cells expressing TEL-JAK2 or Jak2 V617F, respectively, bearing the indicated mutation were diluted into medium (as above) containing the drug to a total volume of 100 µL/well. Each cell line and mutation was represented in triplicate, with the values averaged for plotting and statistical analysis. Cells were incubated in drug for 48 hours at 37°C. Post-48 hour incubation, 25 µL of pre-warmed XTT solution (diluted in medium: 125 nM Phenazine methosulfate (Sigma); 930 µM XTT (BioVectra; Charlottetown, PE)) was added to each well, and cells were incubated at 37°C for an additional eight hours. Absorbance at 450 nm was determined using a 96-well plate spectrophotometer (Molecular Devices Optimax Turntable Microplate Reader).

### Protein Structural Analysis

The JAK2 kinase domain structure complexed with JAK Inhibitor-I was obtained through the protein data bank (http://www.rcsb.org/, PDB ID: 2B7A). Structural analysis and image rendering was performed with PyMOL (http://www.pymol.org/).

### Statistical Analyses

Data are expressed as mean +/− SD. All graphs were generated with and the two-way ANOVA with Bonferroni’s post test was performed, using GraphPad Prism version 5.0 c for Mac OS X, GraphPad Software, San Diego California USA, www.graphpad.com.

## Results

### BaF3 Cells Transduced with a Mutagenized TEL-JAK2 Library Identify Inhibitor-resistant JAK2 Kinase Domain Mutants

**Figure 1:**
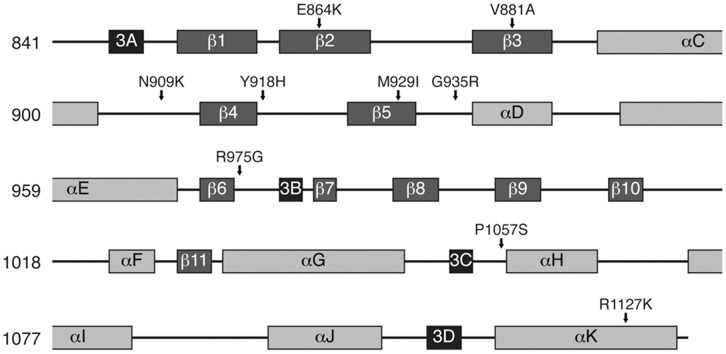
Location of the putative JAK2 inhibitor-resistant mutations. The JAK2 kinase domain secondary structure is labeled as previously published [47], [55]. Residues 841 to 1132 are displayed. Mutations from Table 1 are displayed mapped to the JAK2 residue numbers. Identified mutations do not preferentially map to structured or unstructured regions. Beta sheets (β), alpha helices (α), and 3_10_ alpha helices (3A, 3B, 3C, 3D) are labeled [55].

XL1-Red competent *E. coli* were transformed with pMPG2-TEL-JAK2(5-12), producing a mutagenized library. TEL-JAK2(5-12), referred to as TEL-JAK2, contains the PNT oligimerization domain of TEL and the kinase and pseudokinase domains of JAK2. BaF3 cells were transduced with the mutagenized TEL-JAK2 library and incubated in soft agar containing 1.93 µM JAK Inhibitor-I. Colonies presumably expressing mutagenized TEL-JAK2 were observed on the plates. One hundred colonies were selected, expanded, and both the kinase domain and pseudo-kinase domain were sequenced. Nine kinase domain mutations (Table 1) and zero pseudo-kinase domain mutations were identified. For ease of interpretation, the wild-type human JAK2 amino acid numbering is used. For TEL-JAK2 mutant residues, please see Table S1. Kinase domain mutations were identified once each in the screen. When mapped on the secondary structure of human JAK2, we do not observe clustering within our panel of mutations (Figure 1). Four mutations lie within secondary structure including β2, β3, and the hinge region. Five mutations lie within unstructured regions. The BCR-ABL T315I mutation lies within the ABL kinase domain hinge region, so we generated the homologous mutation in JAK2 (M929I, Figure 1) to determine whether it confers inhibitor resistance. Thus, we have optimized a soft-agar assay to identify inhibitor-resistant mutations in the JAK2 kinase domain.

**Table 1:** Isolated TEL-JAK2 mutations identified in a soft agar screen.

G831R
E864K
V881A
N909K
Y918H
M929I*
G935R
R975G
P1057S
R1127K

BaF3 cells were transduced with a TEL-JAK2 mutant library and incubated in soft agar containing 1.96 µM JAK Inhibitor-I that caused apoptosis in wild-type cells. Colonies capable of growth were expanded and the kinase domain of TEL-JAK2 was sequenced. Each mutation listed was identified once in the screen. Mutation numbering is in the context of human JAK2, despite a different amino-terminus of TEL-JAK2. M929I was generated independently of the screen (indicated by asterisk), modeling the T315I gatekeeper mutation found in inhibitor-resistant BCR-ABL. Correct TEL-JAK2 residue identification can be seen in Table S1.

### TEL-JAK2 Kinase Domain Mutations are Sufficient to Support Growth and Downstream Signaling at High Concentrations of JAK Inhibitor-I

**Figure 2:**
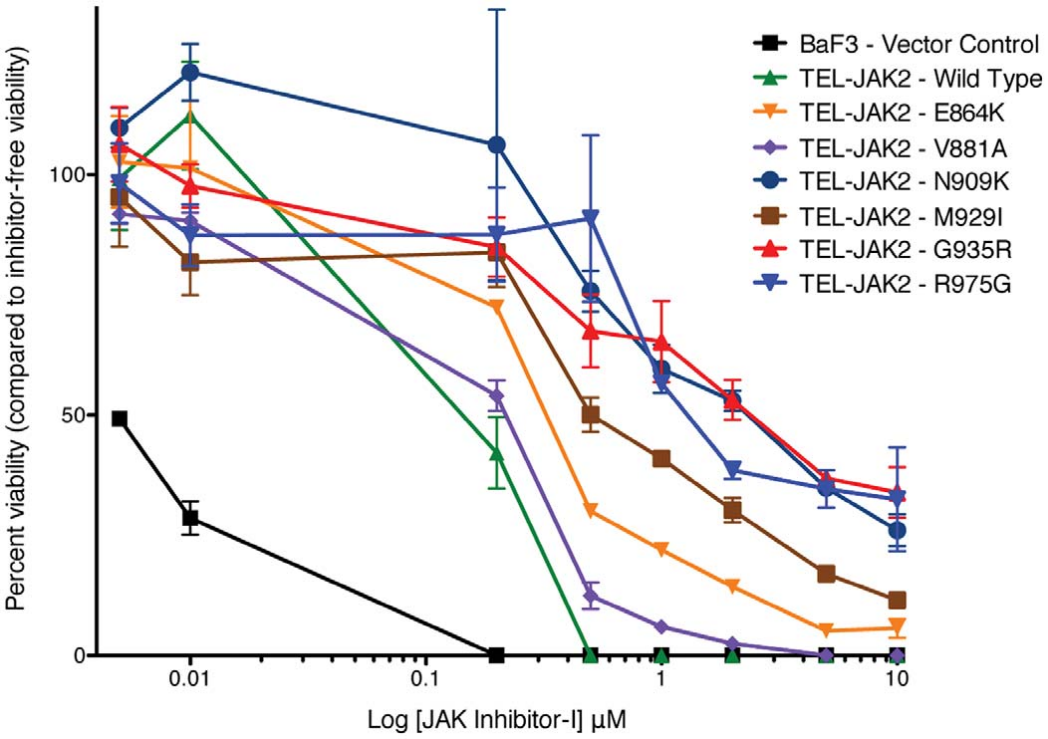
JAK2 mutations display resistance to JAK Inhibitor-I. BaF3 hematopoietic cells expressing the construct indicated were treated for 48 hours in cytokine-free medium containing two-fold increasing concentrations of JAK Inhibitor-I. Cell viability was determined using the XTT assay. The figure is representative of three independent experiments.

**Figure 3:**
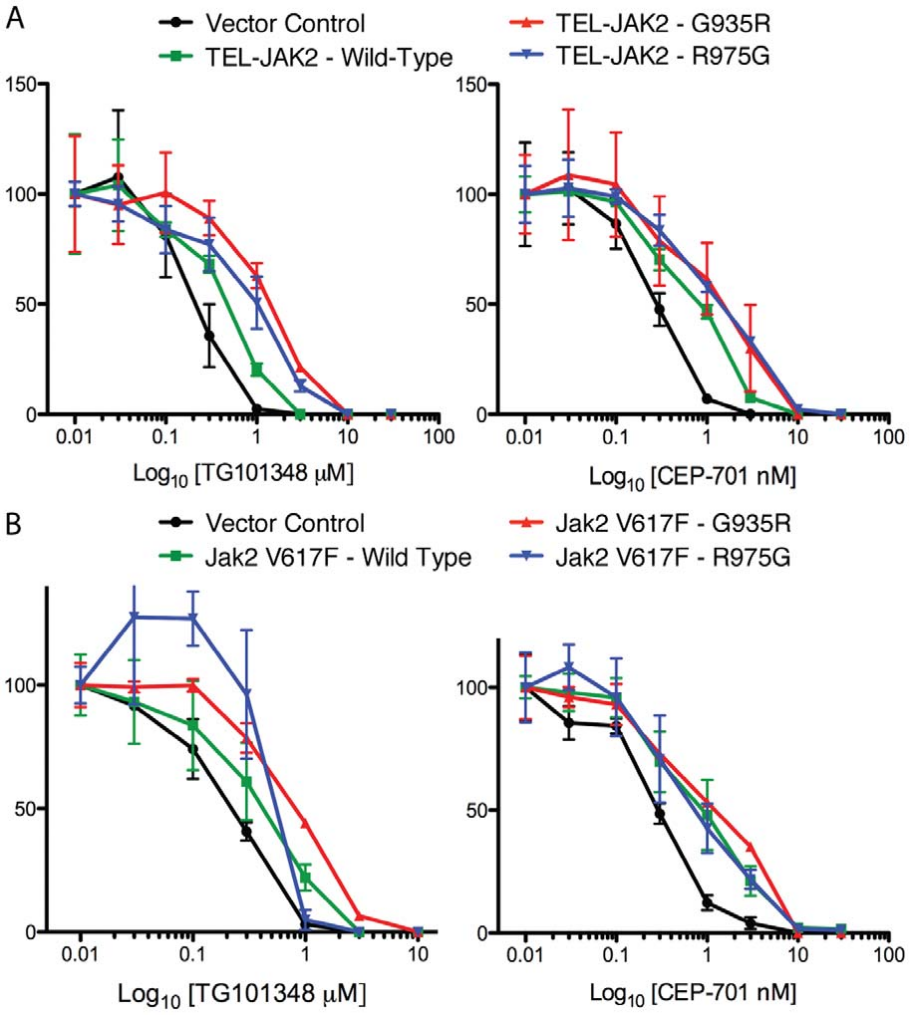
TEL-JAK2 and Jak2 V617F mutants are not resistant to TG101348 or CEP-701. (A) BaF3 hematopoietic cells expressing the TEL-JAK2 construct indicated were treated for 48 hours in cytokine-free medium containing 0.01–30 µM of the indicated inhibitor. Cell viability was determined using the XTT assay. (B) BaF3 EPO-R hematopoietic cells expressing the Jak2 V617F construct indicated were treated for 48 hours in cytokine-free medium containing 0.01–30 µM of the indicated inhibitor. Cell viability was determined using the XTT assay. The figure is representative of three independent experiments.

In order to determine if the identified mutations were responsible for the inhibitor resistance and growth in the soft agar system, the mutations were generated in pMPG2-TEL-JAK2 and used to transduce BaF3 cells. An XTT assay was conducted with cells expressing selected mutants and treated with increasing doses of JAK Inhibitor-I to determine whether the identified mutations can support growth in inhibitor. In BaF3 wild-type TEL-JAK2 cells, death was observed at 0.25 µM (Figure 2). In contrast, cells expressing each of the tested mutants grew, albeit at differing abilities, at 0.25 µM. The TEL-JAK2 mutations N909K, G935R, and R975G group together at 0.25 µM JAK Inhibitor-I, and maintain a 40% growth rate at 10 µM, suggesting very strong inhibitor resistance. Interestingly, cells expressing the engineered mutant TEL-JAK2 M929I (homologous to BCR-ABL T315I) were inhibitor resistant but not to the degree of the strongest three mutants isolated in the screen. TEL-JAK2 wild type, G935R, and R975G were also examined by XTT in the presence of TG101348 and CEP-701 (Figure 3A). A statistically significant difference in growth between wild type and mutants of TEL-JAK2 was not observed with either inhibitor.

**Figure 4:**
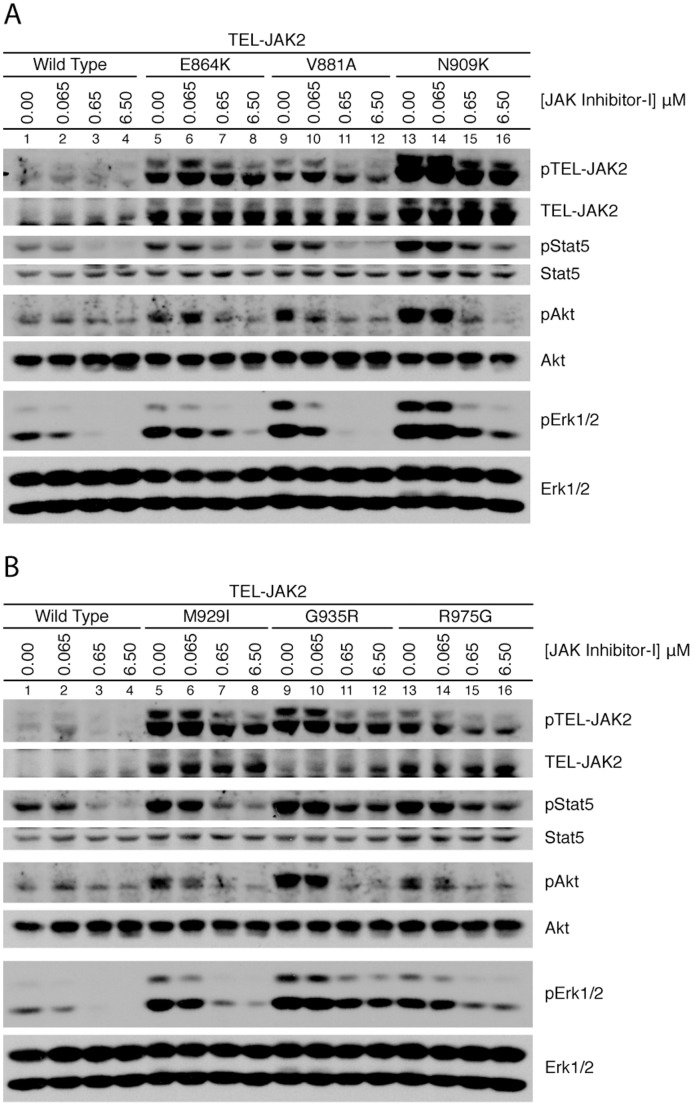
TEL-JAK2 inhibitor-resistant mutants display enhanced phosphorylation of Stat5, Akt and Erk1/2. The indicated BaF3 TEL-JAK2 mutant cell lines were cultured in RPMI complete medium containing increasing concentrations of JAK Inhibitor-I for four hours. Lysates were isolated and JAK2, Stat5, Akt, and Erk1/2 phosphorylation and expression was assessed by immunoblot. The figure is representative of four independent experiments.

Next we investigated the intracellular signaling downstream of TEL-JAK2. We probed for TEL-JAK2, Stat5, Akt, and Erk1/2 phosphorylation (Figure 4A and 4B). Enhanced TEL-JAK2 phosphorylation was observed when inhibitor-resistant mutations were incubated in JAK Inhibitor-I, compared to wild-type TEL-JAK2. Variable expression of TEL-JAK2 was observed with some mutants. TEL-JAK2 wild-type subclones displaying variable total expression were isolated and displayed no significant difference in overall survival (data not shown), suggesting total TEL-JAK2 expression does not correlate with survival ability. Substantially stronger Stat5 activation was observed in all mutants, when compared to wild type, at all tested concentrations of inhibitor. Enhanced Akt phosphorylation was observed in all TEL-JAK2 mutants in the presence of JAK Inhibitor-I, suggesting that Akt activation is coupled to enhanced cell survival in the presence of inhibitor. Erk1/2 phosphorylation was observed at higher concentrations of inhibitor, particularly in cells expressing TEL-JAK2 E864K (Figure 4A, lanes 5–8), N909K (lanes 9–12), G935R (Figure 4B, lanes 9–12), and R975G (lanes 13–16). These results suggest we have identified a panel of JAK2 kinase domain mutants that can sustain growth in high concentrations of inhibitor, perhaps due to activation of Stat5 and Erk1/2 anti-apoptosis or survival pathways.

### Specific TEL-JAK2 Kinase Domain Mutations can Support Elevated Kinase Activity at High Inhibitor Concentrations

**Figure 5:**
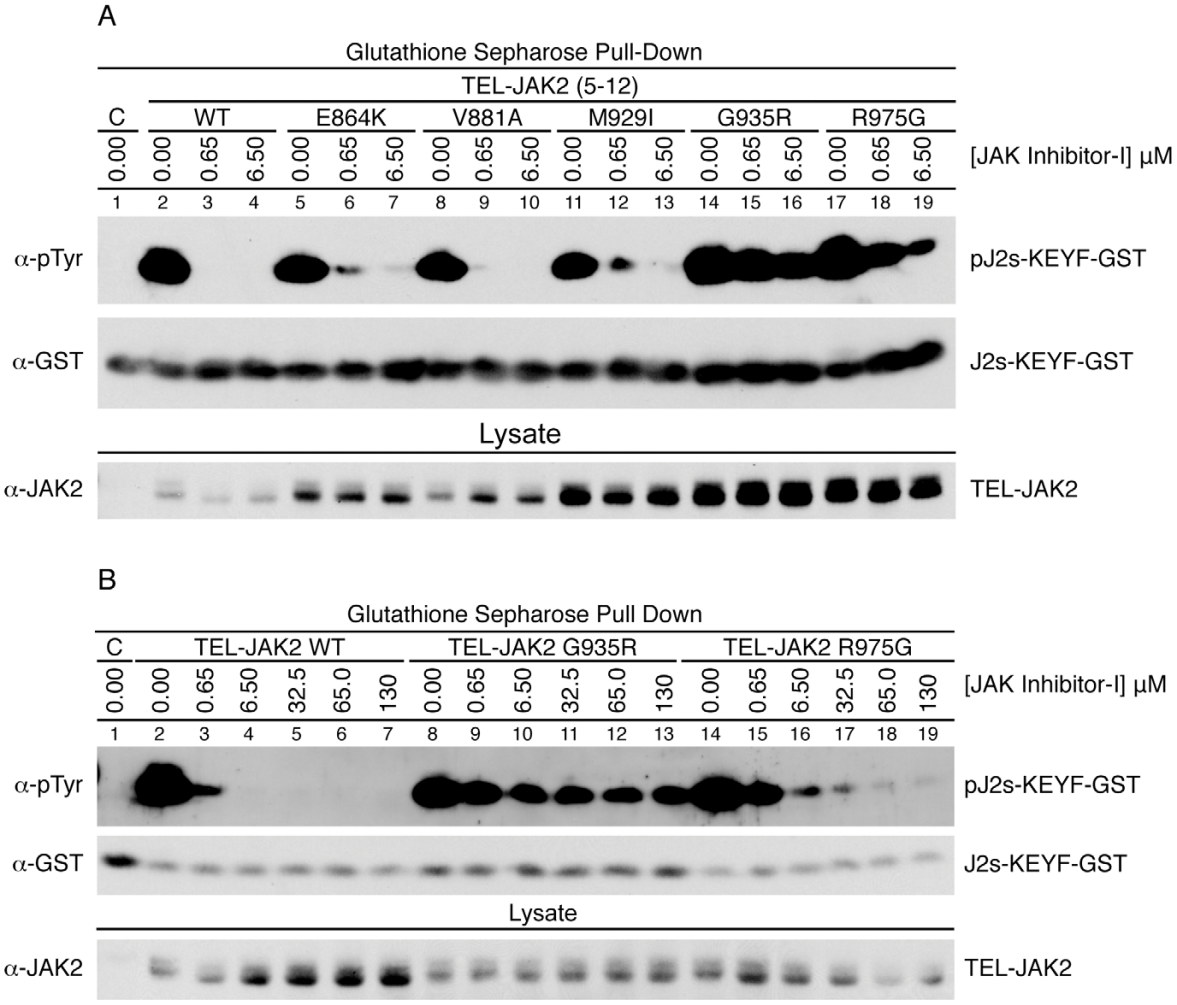
TEL-JAK2 mutants G935R and R975G display a strong degree of inhibitor resistance. 293T cells were co-transfected with the TEL-JAK2 construct indicated and a GST-JAK2 substrate fusion gene (GST-J2s-KEYF). Post-transfection, cells were incubated with the indicated JAK Inhibitor-I concentration for four hours. Cells were lysed and the GST fusion protein was isolated. Phosphorylation of the JAK2 substrate and the TEL-JAK2 fusion protein were assessed with a phospho-tyrosine specific antibody. Total GST and total TEL-JAK2 were also examined. (A) JAK2 substrate phosphorylation was assessed between TEL-JAK2 wild type, E864K, V881A, M939I, G935R, and R975G at three concentrations of inhibitor. The figure is representative of three independent experiments. (B) JAK2 substrate phosphorylation was assessed between TEL-JAK2 wild type, G935R, and R975G at seven inhibitor concentrations. Five micrograms of TEL-JAK2 wild type, and 1 µg of both G935R and R975G was transfected. Phosphorylation of the JAK2 substrate is observed at 0.65 µM inhibitor due to a longer immunoblot exposure, as compared to panel A. The figure is representative of three independent experiments.

To investigate the ability of the TEL-JAK2 mutants to function as kinases in high concentrations of inhibitor, we designed a JAK2 substrate fusion protein combining the glutathione S-transferase protein with an 11 amino acid sequence modeling the JAK2 activation loop [36] (PQDKEYYKVKE, referred to as GST-J2s-KEYY). Three additional constructs were generated as controls: PQDKEYFKVKE (GST-J2s-KEYF), PQDKEFYKVKE (GST-J2s-KEFY), and PQDKEFFKVKE (GST-J2s-KEFF). 293T cells were transfected with pMPG2-TEL-JAK2 and one of the four JAK2 substrate variants in order to assess the ability of TEL-JAK2 to phosphorylate the tyrosines within these substrate fusion proteins (). TEL-JAK2 stimulates tyrosine phosphorylation of a doublet in GST-KEYY (Figure S1B), so GST-KEYF was utilized for intra-cellular kinase assays testing TEL-JAK2 mutants. TEL-JAK2 did not phosphorylate the GST-J2s-KEFF or KEFY proteins (Figure S1A). After substrate optimization, 293T cells expressing pMPG2-TEL-JAK2 and pEBG-GST-J2s-KEYF were incubated with JAK Inhibitor-I for four hours, lysed, the JAK2 substrate fusion protein was isolated with glutathione sepharose beads and probed for phosphorylation (Figure 5A). All tested mutants display phosphorylation of the JAK2 substrate at 0.65 µM, a JAK Inhibitor-I concentration that suppresses wild-type TEL-JAK2 substrate phosphorylation. TEL-JAK2 E864K, V881A, and M929I phosphorylate the substrate slightly at higher JAK Inhibitor-I concentrations. Only TEL-JAK2 G935R (Figure 5A, lanes 14–16) and R975G (lanes 17–19) display substantial kinase activity at 6.5 µM. To test the maximal concentration of inhibitor at which G935R and R975G are able to retain kinase function, we incubated transfected 293T cells in JAK Inhibitor-I up to 130 µM. Wild-type TEL-JAK2 phosphorylation was observed at 0.65 µM JAK Inhibitor-I in a long immunoblot exposure (Figure 5B). TEL-JAK2 G935R retains kinase activity exceeding 130 µM JAK Inhibitor-I (Figure 5B, lanes 8–13), while TEL-JAK2 R975G activity is attenuated but still present (lanes 14–19). Interestingly, in 293T cells TEL-JAK2 expression is variable. This result suggests that the isolated TEL-JAK2 mutations disrupt protein stability or turnover. In order to address this issue, we transfected five-fold more wild-type TEL-JAK2 than G935R and R975G and determined that normalization of TEL-JAK2 expression does not affect its kinase activity at high doses of JAK Inhibitor-I (Figure 5B). These results suggest that selected TEL-JAK2 mutations are at least 200-fold more resistant to JAK Inhibitor-I than wild type.

### Specific Identified Mutations Using TEL-JAK2 Confer Inhibitor Resistance in the Context of Jak2 V617F in both Growth and Downstream Signaling

**Figure 6:**
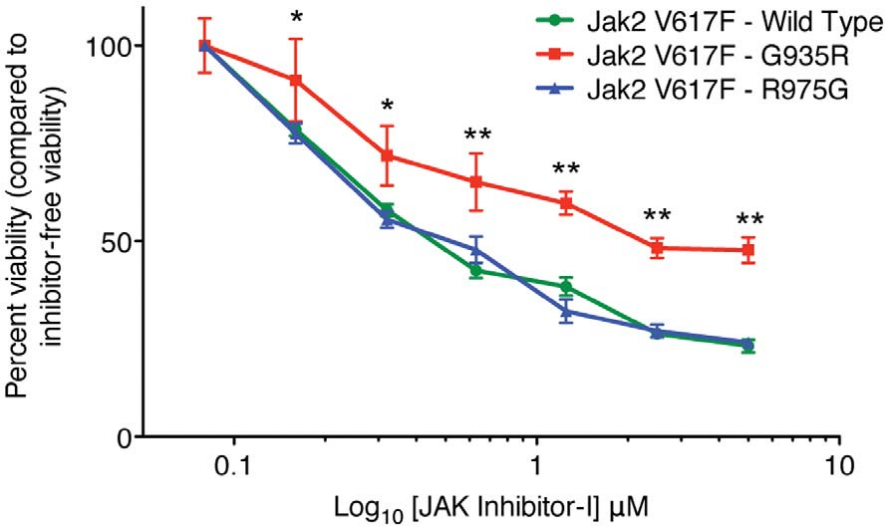
Jak2 V617F G935R is resistant to JAK Inhibitor-I. BaF3 hematopoietic cells expressing the construct indicated were treated for 48 hours in RPMI medium plus 0.1 units/mL EPO containing two-fold increasing concentrations of JAK Inhibitor-I. Post-treatment cell viability was determined using the XTT assay. *: p<0.01; **: p<0.001 for Jak2 V617F G935R compared with Jak2 V617F (two-way ANOVA followed by Bonferroni’s post test). Concentrations up to 30 µM tested and cell lines display the same trend. The figure is representative of three independent experiments.

The initial soft agar screen was completed with mutagenized TEL-JAK2. We hypothesized that, due to the identity between the kinase domains of TEL-JAK2 and Jak2 V617F, any inhibitor-resistant mutation discovered in TEL-JAK2 would be directly transferrable to Jak2 V617F. The panel of TEL-JAK2 mutations was generated in the homologous residues of Jak2 V617F in order to test this hypothesis. BaF3 EPO-R cell lines were generated by transducing cells with one of the panel of Jak2 V617F mutants. We chose the BaF3 EPO-R cell line because it has been demonstrated that Jak2 V617F requires a cytokine receptor scaffold to function [9] and consequently display inhibitor resistance. As predicted, Jak2 V617F wild-type and mutant cells displayed no difference in growth in JAK Inhibitor-I when incubated in the absence of EPO in an XTT growth assay (data not shown). To test the growth ability of our most inhibitor-resistant mutations, we conducted an XTT assay in 0.1 unit/mL EPO plus increasing concentrations of JAK Inhibitor-I. A statistically significant difference in growth between wild-type Jak2 V617F and Jak2 V617F G935R was observed at a JAK Inhibitor-I concentration of 1.25 µM and higher (Figure 6). However, we did not observe a growth difference between Jak2 V617F wild type and R975G. Jak2 V617F G935R, and R975G were also examined by XTT in the presence of TG101348 and CEP-701 (Figure 3B). A statistically significant difference in growth was not observed.

**Figure 7:**
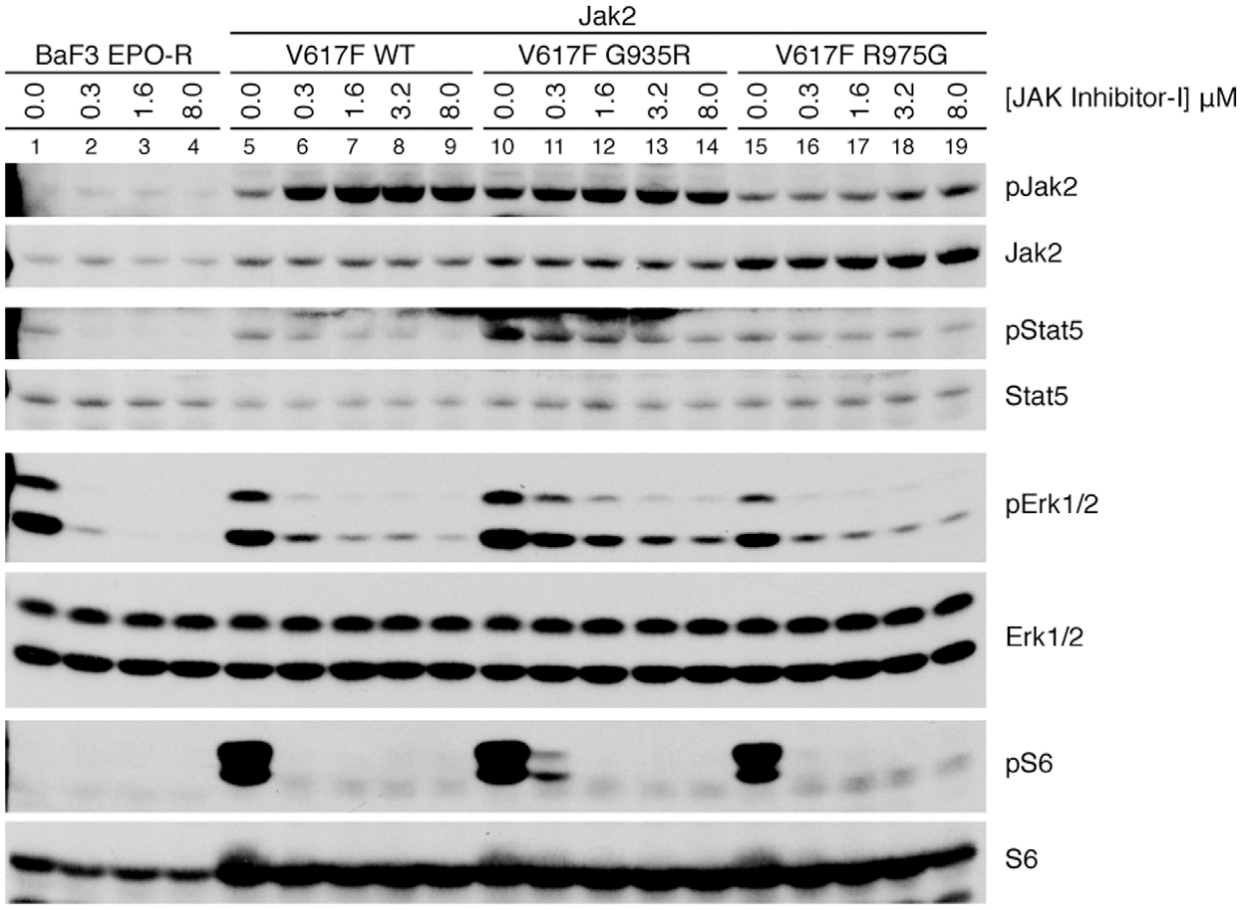
Jak2 V617F G935R displays enhanced Stat5 and Erk1/2 phosphorylation. BaF3 EPO-R cells expressing Jak2 V617F G935R and R975G were cultured in RPMI complete medium containing 0.1 units/mL EPO and increasing concentrations of JAK Inhibitor-I for four hours. Lysates were isolated and Jak2, Stat5, Akt, and ribosomal S6 kinase phosphorylation and expression were assessed by immunoblot using anti-phospho and anti-total antibodies, respectively. The figure is representative of three independent experiments.

Next, the intracellular signaling downstream of Jak2 V617F was investigated. We probed for Stat5, Erk1/2, and S6 kinase activation (Figure 7). JAK Inhibitor-I silences Stat5 signaling in the BaF3 EPO-R cell line at all concentrations tested, whereas Stat5 phosphorylation in wild-type Jak2 V617F is suppressed at 8.0 µM (Figure 7, lane 9). In contrast, both G935R (lane 14) and R975G (lane 19) show sustained Stat5 phosphorylation up to 8 µM. Erk1/2 phosphorylation in blocked above 1.6 µM JAK Inhibitor-I in BaF3 EPO-R cells. Erk1/2 signaling is also attenuated in wild-type Jak2 V617F and R975G in increasing inhibitor concentrations, but appears to be stronger in G935R. S6 kinase is activated at low concentrations of inhibitor only in G935R. Addition of JAK Inhibitor-I resulted in increased Jak2 phosphorylation in BaF3 EPO-R cells expressing Jak2 V617F. Similar results have been reported previously [37], [38], [39]. These results suggest the survival difference observed between Jak2 V617F wild type and Jak2 V617F G935R may be due to enhanced Erk1/2 activation, or perhaps S6 kinase.

### Inhibitor-resistant Mutations in the Context of JAK2 V617F can Support Kinase Activity at an Inhibitor Concentration more than 30-fold Higher than Wild Type

**Figure 8:**
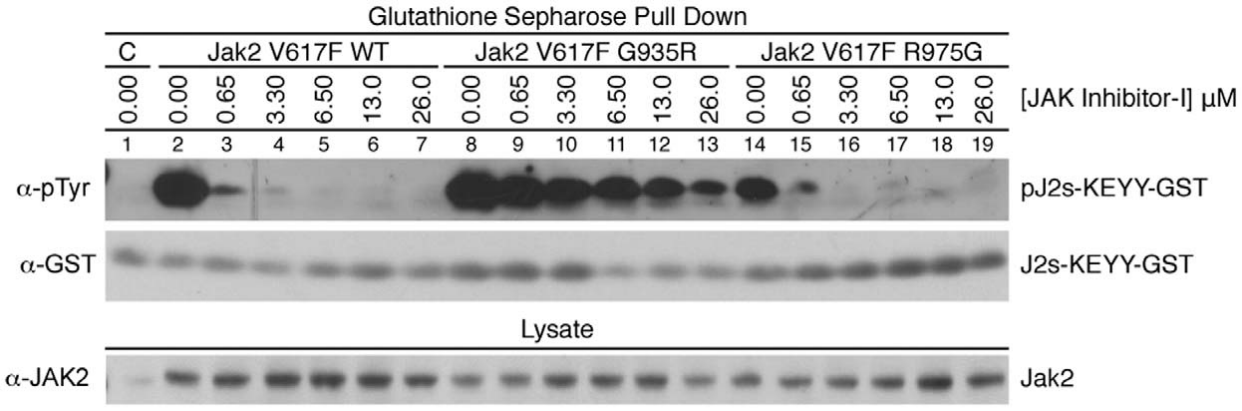
Jak2 V617F G935R displays a strong degree of inhibitor resistance. 293T cells were co-transfected with the TEL-JAK2 construct indicated and a GST-JAK2 substrate fusion gene (GST-J2s-KEYY). Post-transfection, cells were incubated with the indicated JAK Inhibitor-I concentration for four hours. Cells were lysed and the GST fusion protein was isolated. Phosphorylation of the JAK2 substrate and the Jak2 V617F mutant protein were examined by the phospho-tyrosine specific antibody 4G10. Total GST and total Jak2 were also assessed. The figure is representative of three independent experiments.

In order to compare the function of the Jak2 mutant kinase in the context of V617F, we used the JAK2 activation loop (KEYY) GST fusion construct to examine Jak2 kinase activity in the presence of JAK Inhibitor-I. 293T cells were co-transfected with a vector expressing Jak2 V617F wild type, G935R, or R975G, and the GST-J2s fusion vector. Cells were treated with JAK Inhibitor-I for four hours and lysed. The JAK2 substrate protein was isolated with glutathione sepharose beads, and probed for phosphorylation (Figure 8). Jak2 V617F G935R displays very strong kinase function up to 26 µM JAK Inhibitor-I, a 30-fold increase over wild type function. Wild-type Jak2 bearing either G935R or R975G does not phosphorylate the substrate (Figure S1B). Taken together, these data suggest we have identified a mutation in Jak2 V617F that retains significant kinase ability in high concentrations of JAK Inhibitor-I.

## Discussion

Inhibitor resistance is currently one of the biggest challenges facing effective treatment of CML. Evidence suggests that BCR-ABL mutations are present at the commencement of treatment, and the inhibitor provides strong selective pressure for affected clone outgrowth and consequent patient relapse [40], [41]. Considerable effort has been put forth in identifying and testing new generations of inhibitors targeting specific BCR-ABL mutations. The *in vitro* prediction of BCR-ABL mutations against multiple inhibitors was robust and provided the field with significant data to aid in the design of second and third generation kinase inhibitors [22].

Identification of a single point mutation, JAK2 V617F, thought to play an important role in MPN development and progression, initiated the search for small-molecule inhibitors of the JAK2 tyrosine kinase. We hypothesized that inhibitor-resistant JAK2 alleles may become apparent as large cohorts of MPN patients progress through clinical trials testing JAK2-selective drug therapies. The objective of our study was to identify JAK2 mutations that provide resistance to small molecule inhibitors before patient relapse is observed in the clinic.

*TEL-JAK2* is a fusion gene created by the t(9;12)(p24;p13) translocation [42], [43]. The identity between the Jak2 and TEL-JAK2 kinase domains has allowed us to directly apply findings in TEL-JAK2 to Jak2 V617F. BaF3 cells expressing each mutation in TEL-JAK2 were evaluated with an XTT assay to indirectly determine growth in the presence of inhibitor. TEL-JAK2 N909K, G935R, and R975G cluster very closely together in their survival profile, followed by M929I, E864K, and V881A. This result is closely mirrored in the signaling data in which TEL-JAK2 N909K (Figure 4A), G935R, and R975G (Figure 4B) have similar pStat5, pAkt and pErk1/2 activation at higher inhibitor concentrations. The weakest mutant, TEL-JAK2 V881A, survives slightly better than wild type at 0.25 µM JAK Inhibitor-I, and the minor difference is evident when comparing wild type and V881A signaling profiles. Some variation in the activation of Stat5, Akt and Erk1/2 was observed in the absence of inhibitors with the inhibitor-resistant mutants. TEL-JAK2 mutants with elevated basal phosphorylation of downstream signaling components correlated with lower *in vitro* kinase activity. For example, TEL-JAK2 V881A had high Erk2 phosphorylation in the absence of JAK Inhibitor-I, but weak kinase activity upon drug addition. We also examined growth ability in the presence of two clinically relevant inhibitors, TG101348 and CEP-701 (Figure 3B). The lack of growth difference observed in the XTT data suggests we have isolated compound-specific, not ATP competitor-specific, mutations.

To further understand how the JAK2 kinase domain has been modified by the presence of mutations, we developed a novel intra-cellular assay to directly assess its phosphorylation ability in a system more relevant than a standard *in vitro* kinase assay. By fusing a glutathione S-transferase gene to the JAK2 activation loop, we are able to isolate and directly probe for JAK2 phosphorylation of a *bona fide* JAK2 substrate [36]. Our results confirm the XTT and BaF3 TEL-JAK2 signaling data. Wild-type TEL-JAK2 kinase ability is not detectable at 0.65 µM JAK Inhibitor-I. TEL-JAK2 V881A, E864K, and M929I have a small level of phosphorylation, while G935R and R975G have elevated kinase activity up to 6.5 µM.

Interestingly, some of the identified mutations in TEL-JAK2 did not translate to resistance in Jak2 V617F. We evaluated the entire panel of mutations in the context of Jak2 V617F with XTT-based survival, downstream signaling, and with the GST-J2s kinase assay (data not shown). We observed only JAK2 V617F G935R to display a striking difference in survival, downstream signaling, and substrate phosphorylation in comparison to the wild-type protein and other mutants. There are at least two possible explanations for this finding. First, the difference may be due to the relative kinase strength of TEL-JAK2 compared to Jak2 V617F. The Jak2 V617F allele is not transforming unless it has a functional FERM domain and is provided with a cytokine scaffold [44], and even then is relatively indolent without other mutations present [1], [45], [46]. In contrast, TEL-JAK2 is a potent oncogene, thought to be causative in some cases of acute myeloid leukemia [42], [43]. Therefore, even small differences in inhibitor resistance will be evident with TEL-JAK2, while the homologous mutations may have subtle effects in the context of Jak2 V617F. Second, the mechanisms of activation of TEL-JAK2 and Jak2 V617F are different. The PNT dimerization domain of TEL causes oligimerization of the TEL-JAK2 protein and constitutive activation. Therefore, the inhibitor resistance observed in some TEL-JAK2 mutations may be due to the oligimerization-specific interaction between the kinase domains.

**Figure 9:**
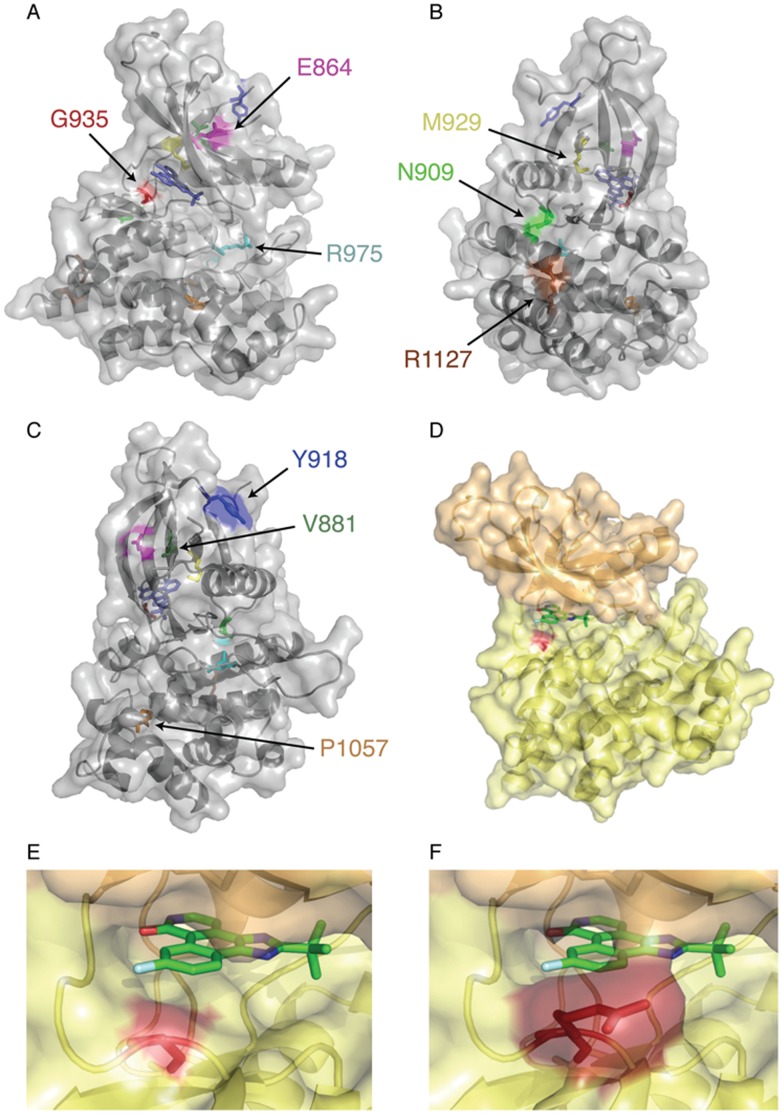
JAK2 inhibitor-resistant residues mapped to the crystal structure bound to JAK Inhibitor-I. The JAK2 kinase domain in complex with JAK Inhibitor-I has been previously published [47]. (A) The face of the kinase domain displays three of the nine residues with identified mutations: E864, G935, R975. (B) Rotated 90 degrees counter-clockwise, three more residues are visible: N909, M929, R1127. (C) Rotated 90 degrees clockwise from 9a are the final three residues: V881, Y918, P1057. (D) The kinase domain is displayed, the n-lobe in orange and c-lobe in yellow. G935 is displayed in red. (E) The kinase domain binding pocket is displayed, G935 in red. (F) The kinase domain binding pocket is displayed, R935 mutation is in red.

In order to understand how the panel of identified mutations contributes to inhibitor resistance, mutations were modeled using the previously published JAK2 kinase domain crystal structure complexed with JAK Inhibitor-I [47] (Figure 9). The unmutated kinase domain residues isolated in the screen are displayed (Figure 9A–C). G935 lies within the hinge region between the N-lobe and C-lobe. The G935R mutation introduces a spatial clash resulting from the arginine side chain, which prevents inhibitor binding (Figure 9D–F). R975 is located in the catalytic loop region connecting α-helix D with the activation loop. The replacement of arginine by glycine (R975G), combined with increased flexibility of the main chain, would influence inter-loop interactions, possibly affecting opening of the pocket. E864K results in a change in side chain charge, and would result in a steric clash with a neighboring lysine. This would result in movement of the β-sheet and occlusion of the pocket. N909K introduces a steric clash that may push neighboring V911 into the binding pocket. The V881A mutation will result in loss of the valine in the hydrophobic core, thereby affecting packing and orientation.

A recent publication has identified activating JAK1 mutations selected for by cytokine deprivation [48]. Interestingly, some of these mutations also confer resistance to the JAK inhibitors CMP6 (JAK Inhibitor-I) and ruxolitinib (INCB018424). In order to compare findings, the murine Jak1 and human JAK2 kinase domains were aligned and the relevant mutations highlighted (Figure S2). Notably, the JAK2 mutations E864K and V881A from this study cluster with the JAK1 mutations D895H, E897K, T901R, and L910Q in the β2 and β3 loop. The strongest mutation in the context of Jak2 V617F, G935R, clusters quite closely with the Jak1 mutation F958V/C/S/L and P960T/S in the kinase domain activation loop. This strong overlap suggests there are common regions in the JAK kinases that are susceptible to mutations that confer inhibitor resistance.

Two recent publications utilized a similar approach as this study: using mutagenesis of Jak2 V617F and incubation with ruxolitinib [49] and mutagenized Jak2 R683G co-expressed with the Crlf2 receptor in BaF3 cells exposed to the BVB808 JAK2 inhibitor [50]. The results of these mutagenesis screens have also been mapped on the mJak1/hJAK2 alignment (Figure S2). In sum, these studies discovered ten inhibitor-resistant mutations that cluster around the ATP-binding pocket. G935R was identified in all three groups, suggesting that G935 lies at a critical interface for inhibitor binding (Figure 9). Weigert *et al.* demonstrated that G935R displayed broad inhibitor-resistance using a wide panel of JAK2-selective inhibitors. Similarly, Y931C (homologous to Jak1 J958 identified by Hornakova *et al*. [48]) was isolated by both the Sattler and Weinstock groups [49]–[50], displayed broad inhibitor resistance. In contrast, the E864K mutation (isolated in this study and by Weigert et al. [50]) displayed narrow inhibitor resistance, suggesting that E864 is more inhibitor specific. The importance of the gatekeeper residue, M929, in Jak2 was verified by Deshpande *et al.* and our study, as the M929I mutation displayed resistance to JAK Inhibitor-1 and ruxolitinib [49]. Other mutations were uniquely identified as resistant to JAK Inhibitor-I (V881A, N909K and R975G) or ruxolitinib (R938L, I960V and E985K) and may represent inhibitor-specific mutations. It is significant to note that all inhibitor-resistant mutations were identified in the Jak2 kinase domain and no allosteric mutations were isolated in the Jak2 pseudokinase or FERM domains. While our approach was a proof-of-concept screen that was not completed to saturation, there is considerable redundancy amongst the three reports, suggesting that fewer Jak2 residues may be critical in mediating inhibitor resistance when compared to the published BCR-ABL studies.

Other JAKs have been targeted by small molecule inhibitors in the treatment of human disease. Inhibition of JAK3 has been explored as an alternative therapy to cyclosporine in transplant rejection and in treatment of rheumatoid arthritis, psoriasis, ulcerative colitis, Crohn’s disease, and dry eye syndrome [51]. Promising clinical trial data have been observed for Tasocitinib (CP690, 550) [52] and VX-509 [53]. In addition, Tasocitinib was also shown to be effective in inhibition of JAK3 and STAT5 activation in peripheral blood mononuclear cells isolated from T-cell leukemia and HTLV-associated myelopathy/tropical spastic paraparesis [54]. The possibility of inhibitor resistance to these agents must not be overlooked.

Our initial *in vitro* results outline a framework to identify and test JAK2 alleles capable of small-molecule inhibitor resistance. Our choice of inhibitor was based on its commercial availability and the published structure complexed with the JAK2 kinase domain [47]. However, our colony selection scheme and evaluation experiments can be applied to any JAK2 inhibitor available. Applied in a high-throughput manner, this experimental procedure may help identify inhibitor-resistant JAK2 mutations before they are observed in the clinic, and therefore allow the development of next-generation inhibitors.

## Supporting Information

Figure S1 **TEL-JAK2 phosphorylates JAK2 substrate activation loop sequences KEYY and KEYF. (**A) 293T cells were transfected with TEL-JAK2 or empty vector and various GST-JAK2 substrate constructs, as indicated. Forty-eight hours post-transfection, cells were lysed, GST fusions were captured on glutathione-sepharose beads, and immunoblotting was performed with anti-phosphotyrosine or GST antibodies. KEXX denotes either KEYY, KEYF, KEFY, or KEFF GST-JAK2 substrate fusion constructs (GST-J2s). The phosphorylation of TEL-JAK2 was confirmed by immunoblotting with an anti-phosphotyrosine antibody. (B) 293T cells were transfected with wild-type Jak2, Jak2 V617F or TEL-JAK2 expression vectors. G935R or R975G mutations were introduced on to each Jak2 backbone. JAK2 expression vectors were co-transfected with GST-J2s vectors as shown. Post-lysis, GST fusion proteins were isolated by glutathione-sepharose and immunoblotting was performed with anti-phosphotyrosine or anti-GST antibodies. Expression of Jak2 constructs was confirmed by performing an immunoblot with an anti-Jak2 antibody.
(TIF)

Figure S2 **Human JAK2 and murine Jak1 domain alignment demonstrate clustering of activating/inhibitor-resistant mutations discovered in separate screens.** Results from Hornakova *et al.* [48] (blue, numbering in reference to mouse Jak1), Deshpande *et al.* [49] (green, numbering in reference to human JAK2), overlayed with our screened mutations (red) suggest clustering within important secondary and tertiary structures in the JAK kinase domain. M929I* denotes an engineered mutation, mimicking the BCR-ABL T315I gatekeeper mutation. E864K, Y931C, and G935R were also reported by Weigert *et al*. [50].
(TIF)

Table S1 **Isolated TEL-JAK2 mutations identified in a soft agar screen, with both TEL-JAK2 and Jak2 amino acid numbering**. Mutations are indicated on the full-length Jak2 backbone. The precise location of each mutation in TEL-JAK2(5-12) is indicated for reference. The asterisk denotes an engineered mutation (M929 homologous to T315 in BCR-ABL).
(DOC)
