# Supplementary figures and images for: Random Mutagenesis Reveals Residues of JAK2 Critical in Evading Inhibition by a Tyrosine Kinase Inhibitor
Source: PLoS One. 2012 Aug 16;7(8):e43437. doi: 10.1371/journal.pone.0043437 (PMC3420867; doi:10.1371/journal.pone.0043437)

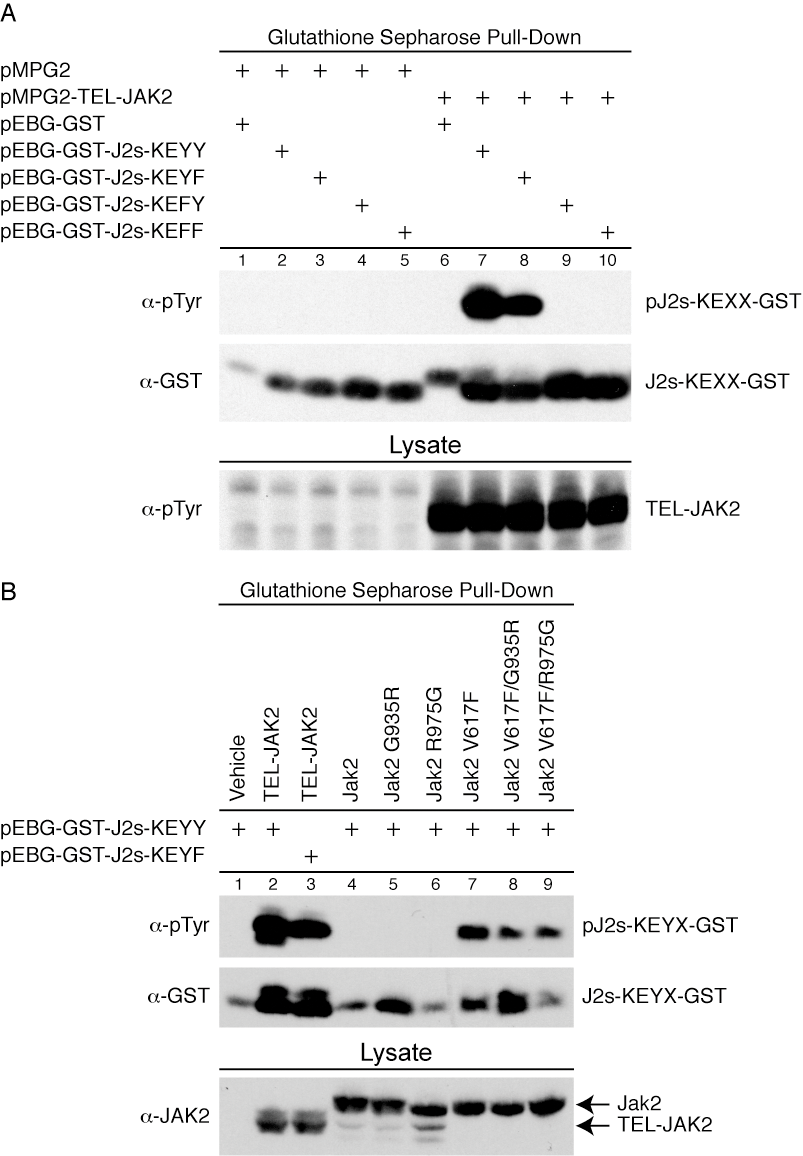

Supplement: Figure S1 — TEL-JAK2 phosphorylates JAK2 substrate activation loop sequences KEYY and KEYF. (A) 293T cells were transfected with TEL-JAK2 or empty vector and various GST-JAK2 substrate constructs, as indicated. Forty-eight hours post-transfection, cells were lysed, GST fusions were captured on glutathione-sepharose beads, and immunoblotting was performed with anti-phosphotyrosine or GST antibodies. KEXX denotes either KEYY, KEYF, KEFY, or KEFF GST-JAK2 substrate fusion constructs (GST-J2s). The phosphorylation of TEL-JAK2 was confirmed by immunoblotting with an anti-phosphotyrosine antibody. (B) 293T cells were transfected with wild-type Jak2, Jak2 V617F or TEL-JAK2 expression vectors. G935R or R975G mutations were introduced on to each Jak2 backbone. JAK2 expression vectors were co-transfected with GST-J2s vectors as shown. Post-lysis, GST fusion proteins were isolated by glutathione-sepharose and immunoblotting was performed with anti-phosphotyrosine or anti-GST antibodies. Expression of Jak2 constructs was confirmed by performing an immunoblot with an anti-Jak2 antibody. (TIF) [file pone.0043437.s001.tif]

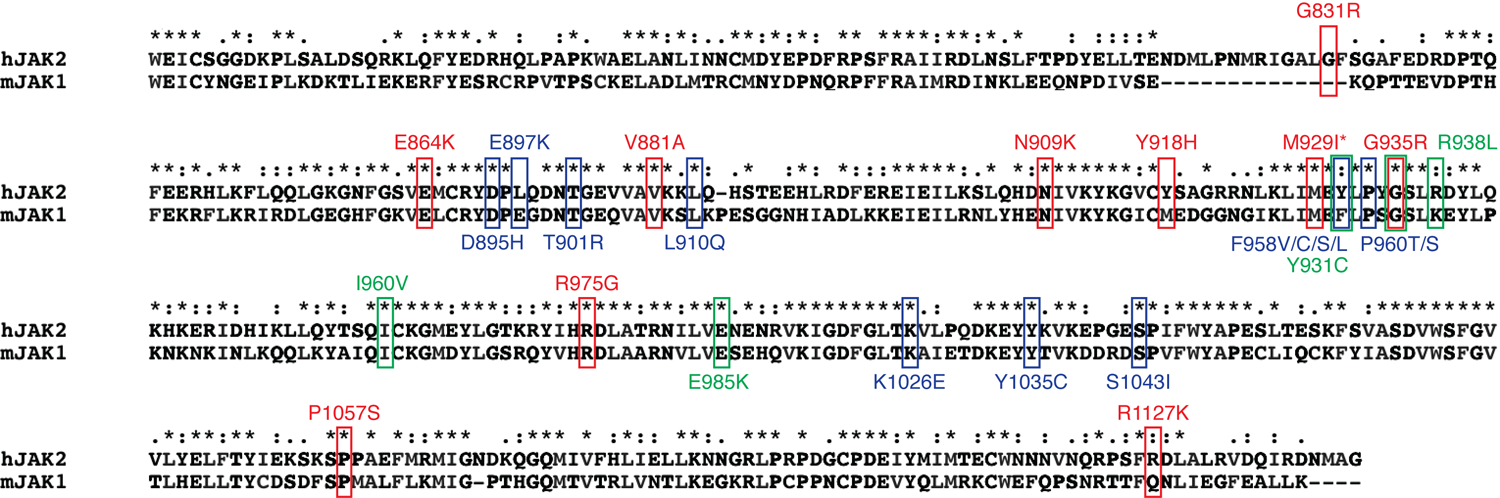

Supplement: Figure S2 — Human JAK2 and murine Jak1 domain alignment demonstrate clustering of activating/inhibitor-resistant mutations discovered in separate screens. Results from Hornakova et al. [48] (blue, numbering in reference to mouse Jak1), Deshpande et al. [49] (green, numbering in reference to human JAK2), overlayed with our screened mutations (red) suggest clustering within important secondary and tertiary structures in the JAK kinase domain. M929I* denotes an engineered mutation, mimicking the BCR-ABL T315I gatekeeper mutation. E864K, Y931C, and G935R were also reported by Weigert et al. [50]. (TIF) [file pone.0043437.s002.tif]
