# Supplementary material for: Random Mutagenesis Reveals Residues of JAK2 Critical in Evading Inhibition by a Tyrosine Kinase Inhibitor
Source: PLoS One. 2012 Aug 16;7(8):e43437. doi: 10.1371/journal.pone.0043437 (PMC3420867; doi:10.1371/journal.pone.0043437)
Supplement: Table S1 — Isolated TEL-JAK2 mutations identified in a soft agar screen, with both TEL-JAK2 and Jak2 amino acid numbering. Mutations are indicated on the full-length Jak2 backbone. The precise location of each mutation in TEL-JAK2(5-12) is indicated for reference. The asterisk denotes an engineered mutation (M929 homologous to T315 in BCR-ABL). (DOC) [file pone.0043437.s003.doc]

| hJAK2 |  | TEL-JAK2 (5-12) |
| --- | --- | --- |
| G831R | = | G663R |
| E864K | = | E696K |
| V881A | = | V713A |
| N909K | = | N741K |
| Y918H | = | Y750H |
| M929I* | = | M761I* |
| G935R | = | G767R |
| R975G | = | R807G |
| P1057S | = | P889S |
| R1127K | = | R959K |

**Supplementary Table 1. Isolated TEL-JAK2 mutations identified in a soft agar screen, with both TEL-JAK2 and Jak2 amino acid numbering**. Mutations are indicated on the full-length Jak2 backbone. The precise location of each mutation in TEL-JAK2(5-12) is indicated for reference. The asterisk denotes an engineered mutation (M929 homologous to T315 in BCR-ABL).
